# Genetic manipulation for inherited neurodegenerative diseases: myth or reality?

**DOI:** 10.1136/bjophthalmol-2015-308329

**Published:** 2016-03-21

**Authors:** Patrick Yu-Wai-Man

**Affiliations:** 1 Wellcome Trust Centre for Mitochondrial Research, Institute of Genetic Medicine, Newcastle University, Newcastle upon Tyne, UK; 2 Newcastle Eye Centre, Royal Victoria Infirmary, Newcastle upon Tyne, UK; 3 NIHR Biomedical Research Centre at Moorfields Eye Hospital and UCL Institute of Ophthalmology, London, UK

**Keywords:** Genetics, Optic Nerve, Retina

## Abstract

Rare genetic diseases affect about 7% of the general population and over 7000 distinct clinical syndromes have been described with the majority being due to single gene defects. This review will provide a critical overview of genetic strategies that are being pioneered to halt or reverse disease progression in inherited neurodegenerative diseases. This field of research covers a vast area and only the most promising treatment paradigms will be discussed with a particular focus on inherited eye diseases, which have paved the way for innovative gene therapy paradigms, and mitochondrial diseases, which are currently generating a lot of debate centred on the bioethics of germline manipulation.

## Introduction

The genetic revolution of the past 25 years has transformed our understanding of the genetic basis of human neurodegenerative diseases.^1^ The causative genes for a large number of well-recognised clinical entities have been identified, and the pace of discovery will only accelerate further as next-generation whole-genome sequencing becomes routinely available to clinicians. In addition to classical monogenic diseases with high penetrance, it is now clear that the majority of common late-onset neurodegenerative disorders are heavily determined by a complex cluster of genetic variants, each contributing to the overall risk of developing overt clinical disease, and in some cases having a synergistic deleterious interaction with environmental triggers.^2^ ^3^ Unfortunately, the significant advances made in deciphering the genetic factors that contribute to the underlying neuropathological process have so far resulted in limited therapeutic benefits for patients. A number of factors have contributed to this frustrating translational gap and the challenges that remain are daunting.

## The burden of disease

The prevalence of monogenic neurological diseases has been estimated at 1 in 1100 in a recent epidemiological study, but when late-onset neurodegenerative disorders, such as Alzheimer disease and Parkinson disease, are included, this figure increases to 1 in 400 of the general population.^4^ This group of disorders is an important cause of chronic morbidity and personal suffering, and it further magnifies the socioeconomic impact of an ageing population by exerting additional stress on already stretched national health services. Treatment remains largely supportive and so far, conventional pharmacological approaches aimed at neuroprotection have failed to deliver effective disease-modifying drugs. To palliate for these inadequacies, genetic manipulation seems an obvious solution as a radical means of correcting the primary pathological process that contributes to neuronal dysfunction and disease progression.

## Gene therapy paradigms

Gene therapy is a priori perfectly suited for ‘single gene’ neurological disorders, but some additional criteria need to be fulfilled, namely: (1) there must be a relatively clear understanding of the pathways contributing to neuronal loss to ensure selection of the most appropriate therapeutic strategy; (2) the natural history of the disease must be clearly defined and it must afford practical windows of opportunity for intervention; (3) the tissue or organ system to be targeted must be relatively accessible; and (4) the specific cells in question must be amenable to efficient transfection with a suitably designed gene vector.^5^ ^6^

Genetic diseases caused by recessive null mutations represent the most straightforward group as the replacement of the missing wild-type protein should prove effective in rescuing the disease phenotype.^7^ Gene therapy for autosomal-dominant disorders can be more challenging if the mechanism is not due to haploinsufficiency, but secondary to a gain-of-function mutant allele that produces an aberrant protein with dominant negative properties.^8^ The mutant protein can either interfere with the wild-type protein to block its normal function or it can have a direct toxic effect on specific cellular processes. In this scenario, simply increasing the production of the wild-type protein with a gene therapy replacement approach might not be able to reverse the negative effect of a dominant gain-of-function mutation. Despite these challenges, Alfred Lewin and colleagues have shown in a mouse model of autosomal-dominant retinitis pigmentosa carrying the P23H *RHO* mutation that increasing the production of normal rhodopsin can suppress the effect of the mutated misfolded protein and prevent photoreceptor degeneration.^9^ However, if increasing the level of the wild-type protein fails to rescue the pathological phenotype for an autosomal-dominant disorder, the most logical strategy is to block the expression of the mutant messenger RNA transcript and supplement the cell with a wild-type copy of the gene if required.^10^ This suppression and replacement approach is technically more complex as it requires a delicate balance of gene expression to be achieved. The main experimental paradigms for gene silencing are based on the use of antisense oligonucleotides, ribozymes or RNA interference.^11–14^

If the causative gene for a rare monogenic disease has not been identified or the mode of inheritance is complex as in late-onset neurodegenerative diseases, gene therapy can still be contemplated as a treatment strategy.^15^ The approach in these situations involve transfecting the cell with gene constructs that upregulate the expression of trophic factors, which in turn serve to rescue neuronal cells from impending death or at least prolong their survival. These blanket neuroprotective strategies could also be used to supplement more targeted gene therapy in monogenic diseases and conceptually, these could provide a synergistic beneficial effect. A completely novel approach for neurodegenerative diseases is optogenetics, which involves the introduction of light-sensitive protein sensors into neurones making them functionally photosensitive.^16^ ^17^ Ion channel proteins of the channelrhodopsin, halorhodopsin and archaerhodopsins families are able to confer these unique properties by modulating neuronal membrane potential and the balance between depolarised and hyperpolarised states. Optogenetics is being used to convert non-photosensitive retinal cells into artificial photoreceptors, and also to deliberately switch on and off specific central nervous system pathways in an attempt to circumvent the damaged circuitry in anatomically diseased areas.^16^ ^17^

## Gene delivery systems

The success of gene therapy is contingent upon an effective delivery system and various vectors have been developed to deliver the genetic construct, which is more commonly DNA, but sometimes RNA.^18^ The use of non-viral vectors has obvious safety advantages as they are devoid of potential immunogenic and neoplastic side effects for the human recipient. Most of these strategies revolve around the use of liposomes and nanoparticles to package the genetic material within a cationic lipid or polymer protective shell.^19^ However, these non-viral delivery systems have limited cargo capacity and therapeutic gene expression is usually low and transient, precluding a sustained therapeutic effect. The favoured alternative is a modified virus that has a natural tropism for the central nervous system and with the ability to integrate genetic material into the host cell's nuclear genome to achieve more prolonged gene expression (table 1). The most commonly used viral vector in human clinical trials, especially for ocular gene therapy, is the adeno-associated virus (AAV).^20^ ^21^ There are now long-term safety data for these recombinant vectors and reassuringly, no major concerns have been raised. AAV vectors are also able to efficiently transduce non-dividing cells, which make them particularly attractive for neuronal populations. A wide variety of AAV serotypes have been genetically engineered by altering the proteins on the outer shell (capsid) and the DNA sequence. These genetic modifications confer specific cellular tropism and they also influence the onset and the intensity of transgene expression. AAV serotype 2 (AAV2) has a natural predilection for retinal cell types and it can induce prolonged levels of gene expression, potentially maximising the intended therapeutic effect. Despite their versatility, AAV vectors have a number of disadvantages including a limited transgene capacity (4.5 kb) and the risk of being rapidly eliminated by the humoral immune response in patients who have previously been exposed to the virus and possessing high circulating levels of neutralising antibodies.^20^ ^21^ As an alternative, lentiviral vectors have gained increasing popularity for central nervous system disorders because of their larger transgene capacity and their ability to sustain high levels of gene expression, although there remains safety concerns with regard to the potential for insertional mutagenesis.^22–24^ Besides the intrinsic properties of the viral carrier, another fundamental aspect of vector design is the use of an appropriate promoter sequence that can efficiently drive the expression of the transgene within the target cell. Depending on the therapeutic aims, the viral vector can be tailor-made with additional promoter elements that titrates the level of gene expression or even limits its action to specific cell types.

**Table 1:** Viral delivery systems for the treatment of neurodegenerative diseases

Characteristics	Adeno-associated virus	Adenovirus	Retrovirus	Lentivirus	Herpes simplex virus
Wild-type virus	Single-stranded DNA (4.7 kb)	Double-stranded linear DNA (36 kb)	Diploid positive strand RNA (9.2 kb)	Diploid positive strand RNA (9.2 kb)	Double-stranded linear DNA (152 kb)
Maximum insert size	4.5 kb	7.5 kb	8.0 kb	8.0 kb	20–40 kb
Achievable titre (per ml)	High (>10^12^)	High (>10^11^)	Low (>10^8^)	Low (>10^8^)	High (>10^9^)
Infectivity	Broad	Broad	Dividing cells	Broad	Broad
Chromosomal integration	No*	No	Yes	Yes	No
Transgene expression	Long (months to years)	Short (days to weeks)	Long (months to years)	Long (months to years)	Short (days to weeks)
Latency in host cells	No	Yes	No	No	Yes
Pre-existing immunity	Yes	Yes	Unlikely	Unlikely†	Yes
Host immunological response	Minimal	Extensive	Minimal	None	Moderate
Potential in vivo risks	Insertional mutagenesis*	Inflammatory response	Insertional mutagenesis	Insertional mutagenesis	Inflammatory response

*Some integration occurs, but at low frequency. The risk of insertional mutagenesis is minimal compared with other viral vectors.

†Except perhaps in patients with HIV infection.

## Retinal neurodegenerative diseases

The eye represents a target organ of choice for gene therapy as it is easily accessible and rather advantageously, it is an immunologically protected space.^5^ It is therefore hardly surprising that inherited retinal diseases have led the way both in terms of pioneering the clinical application of gene therapy and the refinement of the protocols for achieving efficient gene delivery in vivo. Advances in minimally invasive intraocular surgery have also made it possible for ophthalmic surgeons to safely access various retinal layers providing a direct route for the delivery of the gene therapy vector. We will now review some key examples of neurodegenerative disease affecting the retinal pigment epithelium (RPE), photoreceptors, and retinal ganglion cells (RGCs) to illustrate some of the groundbreaking innovations that have been achieved to establish gene therapy as a viable treatment option for a broad range of ocular disorders.

### Correcting gene expression

Leber congenital amaurosis (LCA) is a severe form of inherited blindness that affects at least 1 in 50 000 children.^25^ ^26^ It is genetically heterogeneous and so far, 17 disease-causing genes have been identified that account for about half of all diagnosed cases. One group of patients harbour recessive mutations in the *RPE65* (RPE-specific protein 65 kDa) gene, which encodes for a retinoid isomerase that is expressed almost exclusively within the RPE layer.^27^ ^28^ This specific isomerase is a key component of the visual cycle as it converts all-*trans* retinoid to *11-cis* retinal for the regeneration of visual pigment after exposure to light. LCA associated with *RPE65* is a complex disease in which vision loss results from two pathological mechanisms—dysfunction and degeneration of photoreceptors.^25^ ^26^ The accumulation of all-*trans* retinyl esthers has a toxic effect, causing progressive degeneration of both rod and cone photoreceptors, and resulting in profound visual impairment by early adulthood.^29^ As the disease process is secondary to a lack of the wild-type protein, gene therapy aimed at augmenting *RPE65* gene expression was an obvious therapeutic target. Several preclinical studies were initiated worldwide that substantiated this approach, in particular the seminal study by Jean Bennett and colleagues who in 2001 showed that a subretinal injection of an AAV2 vector containing *RPE65* cDNA could restore visual function in three mutant RPE65−/− Briard dogs within 3 months of them being treated.^30^ ^31^ Additional work in murine models and the establishment of Good Manufacturing Practice for the production of viral vectors led to launch of four independent clinical trials for *RPE65*-LCA in the USA and in Europe. The initial reports confirmed the safety of injecting a bolus of an AAV2-*RPE65* vector in the subretinal space, although some investigators caution against the fluid bleb involving the fovea to minimise the iatrogenic loss of foveal cones and secondary retinal thinning.^32–35^ All the studies showed a modest improvement in a number of visual parameters within the first month of treatment, probably due to the partial reconstitution of the canonical retinoid cycle within the RPE, and some patients performed better in a subjective test of visual mobility. The visual benefit persisted for at least 3 years, but there was ongoing loss of photoreceptors in the treated retina at the same rate as that of the untreated retina, and on longer periods of follow-up, the comparative improvement in retinal function was lost altogether.^36–38^ A number of limitations have been identified from this first wave of human clinical trials that will need to be addressed if a more robust and prolonged treatment response is to be achieved. It is clear that the earlier treatment is initiated the better in terms of photoreceptor rescue, and more efficient transduction of the outer retina will be essential to deliver a more substantial augmentation of RPE65 and sustain the visual cycle.^39^ Finally, although the lack of functional wild-type protein is considered to be a major factor driving retinal degeneration in LCA, correcting *RPE65* gene expression on its own might not be sufficient and additional neuroprotective strategies might have to be provided concurrently.

Another form of inherited retinal degeneration where significant progress has been achieved recently is choroideremia, which has an estimated prevalence of 1 in 50 000.^40^ It is an X-linked disorder caused by null mutations in the *CHM* gene and the lack of the encoded Rab escort protein-1 (REP1) accounts for the neurodegenerative process.^41^ ^42^ Loss of night vision begins in the first decade of life and there is gradual loss of peripheral vision leading to legal blindness by the fifth decade of life. The pathological hallmark of choroideremia is the progressive degeneration of the choriocapillaris, the RPE and the outer retina. Patchy areas of chorioretinal atrophy begin in the mid-periphery of the fundus and the foveal region is spared until the end stages of the disease, affording an attractive window of opportunity for therapeutic intervention.^40^ Nearly all reported cases of choroideremia have been attributed to functionally null *CHM* mutations and because the gene has a relatively small protein coding sequence (1.9 kb), it can easily be packaged in an AAV delivery vector. Based on promising preclinical work that confirmed the effectiveness and safety of an AAV-based strategy for delivering the *CHM* cDNA encoding REP1, Robert MacLaren and colleagues initiated a first-in-man gene therapy trial that recruited six male patients with choroideremia and good central visual acuity of 6/6 or better.^43–45^ Although further work is needed, including longer periods of follow-up, the initial results 6 months postsurgery showed improved rod and cone function based on microperimetry and visual acuity tests.^45^ As for LCA caused by *RPE65* mutations, this choroideremia trial further confirms the huge potential of ocular gene therapy, which could be extended in due course to more common retinal neurodegenerative diseases such as age-related macular degeneration.

The encouraging results obtained for LCA and choroideremia have led to a major surge in interest from industry and increased collaborations with academic groups to fast-track development pipelines. More efficient replacement vectors are currently being developed for a broad range of inherited genetic diseases including other genetic forms of LCA besides *RPE-65*, recessive forms of Stargardt disease caused by *ABCA4* mutations and achromatopsia secondary to *CNGB3* deficiency.^7^ A number of research groups worldwide are also actively working on the development of AAV2-based gene therapy for patients with inherited optic neuropathies.^46^ The two most advanced research programmes are for recessive forms of Wolfram syndrome secondary to *WFS1* mutations and for autosomal-dominant optic atrophy (DOA) caused by *OPA1* mutations that result in haploinsufficiency.^47–50^ Compared with gaining access to RPE cells and photoreceptors, RGCs form the innermost layer of the retina, which obviates the need for a vitrectomy or direct physical manipulation of the retina. The ability to deliver the gene therapy vector with a minimally invasive intravitreal injection procedure provides several advantages, in particular a much lower risk of iatrogenic complications and a faster recovery time for the patient. Unlike inherited retinal diseases, gene therapy vectors for DOA and Wolfram syndrome are still in preclinical phases of development, and a number of technical limitations have been encountered that will need to be resolved, in particular RGC transfection efficiency and achieving the optimal gene replacement dosage.^51^ The latter point is only starting to be fully appreciated as it became apparent that supraphysiological levels of OPA1 can, in fact, have a detrimental effect on RGC function, at least in the mouse model that was studied.^52^

### Allotopic gene expression

Leber hereditary optic neuropathy (LHON) is a mitochondrial DNA (mtDNA) genetic disorder characterised by bilateral severe visual loss secondary to the primary loss of RGCs within the inner retina.^53^ ^54^ The visual prognosis is poor with the majority of patients remaining legally blind with visual acuities worse than 6/60.^55^ About 90% of cases are due to one of three mtDNA point mutations (m.3460G>A, m.11778G>A and m.14484T>C), which affect key complex I subunits of the mitochondrial respiratory chain, resulting in reduced ATP production and increased levels of reactive oxygen species (ROS).^56^ ^57^ There is currently no treatment that has been conclusively shown to reverse the rapid loss of RGCs in the acute phase of LHON, and the management of this disorder remains largely supportive.^58^ The m.11778G>A mutation accounts for 60–70% of cases worldwide and gene therapy is being actively pursued as a therapeutic option for this inherited form of mitochondrial blindness. The two main treatment paradigms are allotopic gene expression and the enhancement of neuronal survival with various trophic factors.

Mitochondria have a double-membrane structure and this physical barrier represents a major challenge for direct gene therapy in LHON. An elegant solution to this problem is allotopic gene expression, which involves transfecting a modified version of the replacement gene into the nuclear genome.^59^ ^60^ The modified protein that is produced has a specific targeting sequence that allows for its efficient import into the mitochondrial compartment. Proof of concept was first demonstrated in mutant LHON cybrids with the preferred viral vector being AAV2.^32^ ^33^ The ability to rescue RGCs and improve visual function was subsequently demonstrated in rodent LHON models expressing a mutant form of the ND4 complex I subunit and replicating the pathological consequences of the m.11778G>A mutation.^61^ ^62^ These promising in vitro and in vivo studies have paved the way for the first clinical trials of allotopic gene expression for patients with LHON harbouring this pathogenic mtDNA mutation (NCT02064569 and NCT02161380). There is still some debate whether the imported wild-type ND4 subunit does integrate into complex I of the mitochondrial respiratory chain to produce a stable and biochemically active unit within the inner mitochondrial membrane.^63^ The results of the ongoing LHON gene therapy trials will hopefully help to answer these controversies and more importantly, whether patients experience a functional visual benefit using the gene delivery vectors that have been engineered to correct for the m.11778G>A mutation.

A complementary and possibly synergistic approach to replacing the defective complex I subunit in LHON is to protect RGCs against the deleterious downstream consequences of disturbed mitochondrial function. Increased ROS levels are considered to be a major factor driving the apoptotic loss of RGCs in LHON.^64^ Superoxide dismutase is a key mediator of the cell's antioxidant defence mechanism and conceptually, increasing the activity of this ROS scavenger should have a beneficial impact on neuronal survival under unfavourable cellular conditions. This principle was demonstrated convincingly in m.11778G>A LHON cybrids with allotopic expression of the *SOD2* gene.^65^ The increased expression of superoxide dismutase resulted in increased cell survival, and future work is now needed to determine whether consolidating antioxidant defences within RGCs could magnify the therapeutic potential of correcting for the mutant complex I subunit in patients with LHON. A radically different strategy is based on the xenotopic expression of Ndi1, an alternative NADH oxidase expressed in yeast (*Saccharomyces cerevisiae*) mitochondria.^66^ ^67^ Ndi1 is a versatile enzyme that can bypass a malfunctioning complex I to restore downstream electron transfer while at the same time suppressing ROS overproduction. Successful rescue of optic nerve degeneration was achieved using the yeast *Ndi1* gene in a rat model of LHON that involved injection of rotenone-loaded microspheres into the optic layer of the rat superior colliculus.^68^

### Enhancing neuronal survival

The correction of the underlying protein deficiency in monogenetic diseases is a logical treatment strategy that has moved beyond the proof-of-concept stage. However, despite the major advances in genomic medicine, the aetiology of a number of rare or ultra-rare genetic syndromes still remain undefined, and the genetic determinants for late-onset, sporadic neurodegenerative diseases such as Parkinson disease have not been sufficiently clarified to allow specific genetic risk factors to be targeted.^69^ Even if the primary genetic trigger or sequence of cellular events that cause neuronal loss have not been defined, gene therapy could still prove a useful tool by enhancing the local expression of neuroprotective molecules that have consistently proven efficacious in preclinical studies. Once more, basic research on retinal dystrophies have paved the way with the identification of several neurotrophic factors, for example, ciliary neurotrophic factor, glial-cell derived neurotrophic factor (GDNF) and brain-derived neurotrophic factor (BDNF), which seem to arrest the pathological process irrespective of the genetic subtype.^70–72^ Neurotrophic factors can diffuse away from the cell from which they are secreted and this important property can be used to extend their therapeutic range, especially for central nervous system disorders. This neuroprotective treatment paradigm still needs to be refined, but encouraging preliminary results have been obtained for Parkinson disease with neurturin (NRTN), a structural and functional analogue of GDNF that protects dopaminergic neurones.^73^ ^74^ In a double-blind phase II trial, 58 patients with advanced Parkinson disease were randomly assigned, in a 2:1 ratio, to receive either a bilateral injection of AAV2-*NRTN* into the putamen or sham surgery.^75^ The primary endpoint was the change from baseline to 12 months in the motor subscore of the unified Parkinson disease rating scale. The treatment was well tolerated and although the primary endpoint did not reach statistical significance, positive results were obtained in a subgroup of patients that had been assessed for up to 18 months. Histopathological analysis performed on the brains of two patients who were treated with AAV2-*NRTN* suggested a possible delay in the transport of NRTN from the putamen to the substantia nigra because of a severely degenerated nigrostriatal tract. To address these possible study limitations, a new clinical trial is currently underway to determine whether direct transgene delivery to the substantia nigra, in addition to a higher dose injected into the putamen, will prove beneficial to patients with advanced Parkinson disease when assessed over a longer follow-up period.

### Optogenetics

**Figure 1:**
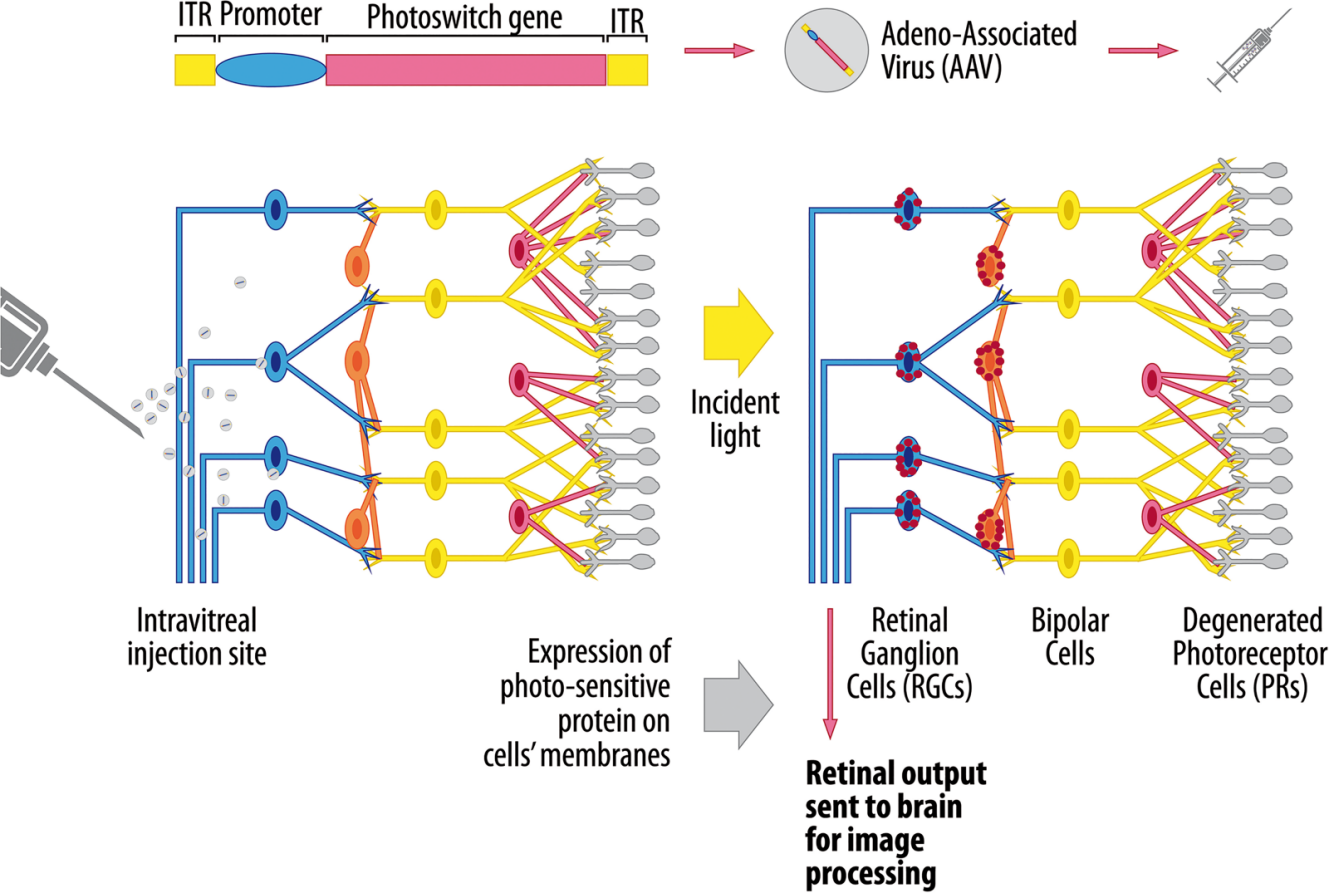
Therapeutic application of optogenetics to retinal neurodegenerative diseases. The gene construct encodes for a photosensitive channel protein belonging to the microbial opsin family and the preferred delivery system is an adeno-associated virus (AAV) vector due to its natural tropism for retinal cells. An intravitreal injection is sufficient when the inner retina is being targeted, whereas delivery of the viral vector into the subretinal space is usually needed to achieve sufficient transfection of outer retinal cells. Cell-specific promoters can be used to limit the expression of the photoswitch gene to specific retinal cell types. The selective expression of the opsin protein is represented by the red dots within retinal ganglion cells (RGCs) and bipolar cells. When stimulated by light of a certain wavelength, the protein channel opens, allowing the redistribution of ions across the cell membrane. The resultant depolarisation or hyperpolarisation of the retinal cells results in the transmission of a visual impulse to the occipital cortex via the optic nerve (http://www.gensight-biologics.com/index.php?page=optogenetics, accessed on 18 December 2015).

Optogenetics is a novel technique that involves imparting light sensitivity onto neurones by transfecting them with bacterial opsin genes encoding for specific ion channel proteins.^76^ ^77^ The opening of these channels is modulated by light and the flux of ions across cell membranes creates an action potential, analogous to a neurone discharging. The attraction of optogenetics for inherited retinal diseases lies in the structured relay system that exists with the mammalian retina, and disorders affecting the outer retina lend themselves particularly well to visual restoration using this technique (figure 1). If there is complete degeneration of the photoreceptor layer, one approach is to render RGCs or bipolar cells light sensitive.^78^ The advantage of targeting bipolar cells is that they are able to generate RGC responses that are physiologically closer to natural activity patterns. A number of studies have shown the efficacy of this method in restoring photosensory responses and visually evoked neural activity in mouse models of retinitis pigmentosa, and rather encouragingly, the treated blind mice showed improved locomotor behaviours.^79–82^ Optogenetics is in an early stage of development and in addition to achieving a sufficient level of transfection, better targeting of transgene expression to specific retinal cells needs to be achieved to avoid unwanted retinal pathways from being activated. Another factor that needs to be considered is the intensity of light and wavelength needed to produce sufficient activation of the opsin-encoded ion channels, but without causing long-term retinal phototoxicity.^78^ The patient will be expected to wear a prosthetic device that delivers the light stimulus to the retina and newer opsin channels with greater photosensitivity are being developed that do not require stimulation with the more damaging blue light spectrum.

The potential applications of optogenetics extend far beyond inherited retinal diseases as the ability to selectively modulate neuronal function could be used to treat central nervous system disorders caused by an imbalance between inhibitory and excitatory pathways.^16^ ^83^ The basal ganglia contain sophisticated neural networks that regulate motor planning and two main pathways with opposing actions have been described.^84^ According to this classical model, activation of the ‘direct’ pathway facilitates movement whereas activation of the ‘indirect’ pathway inhibits movement. The motor dysfunction in patients with Parkinson disease is thought to arise due to a progressive weakening of the direct pathway, driven by the loss of dopamine-secreting nigrostriatal neurones. In a landmark paper, Anatol Kreitzer and colleagues tested this hypothesis in a mouse model of Parkinson disease with optogenic manipulation.^84^ A recombinant AAV1 virus was used to deliver channelrhodopsin-2 and selectively target specific neuronal populations within the basal ganglia. Remarkably, activation of the direct pathway completely rescued deficits in freezing, bradykinesia and locomotor initiation in the mutant mice. Although still in its infancy, these exciting findings have opened up a whole new avenue of translational research for Parkinson disease and other neurodegenerative disorders.^85^

## Mitochondrial neurodegenerative diseases

### Mitochondrial genetics

Mitochondria are ubiquitous organelles present in every nucleated cell in the human body. A unique feature of mitochondria is that they contain their own genetic material in the form of a double-stranded circular DNA molecule, which is about 16 569 bp long.^86^ ^87^ Mitochondrial DNA is a very high-copy number genome with 2–10 mtDNA molecules in each mitochondrion, and hundreds to thousands of mitochondria per cell depending on its overall metabolic expenditure. As a result, two situations can arise, namely homoplasmy or heteroplasmy. In the heteroplasmic state, two or more mtDNA variants are present at a specific nucleotide position, whereas in the homoplasmic, only one mitochondrial allele exists.^88^ Due to its compact size, the mitochondrial genome has limited coding capacity for only 2 ribosomal RNAs, 22 transfer RNAs and 13 essential subunits of the mitochondrial respiratory chain complexes.^86^ ^87^ These encoded gene products are absolutely critical for survival as mitochondria provide for most of the cell's ATP requirements through oxidative phosphorylation. Nevertheless, it is important to remember that the majority of structural and accessory components required for normal mitochondrial function are encoded by the nuclear genome.^89^ This synergistic nuclear–mitochondrial interaction explains why human disease can arise both from mutations in the mitochondrial genome (primary mtDNA disorders) and the nuclear genome (nuclear mitochondrial disorders).^90^

Mitochondrial diseases are now recognised as a major cause of chronic morbidity and the minimum prevalence has been estimated at 1 in 4300 in the general population.^91^ Reflecting the ubiquitous nature of mitochondria and their fundamental roles in energy production, patients with mitochondrial genetic disorders often manifest a heterogeneous combination of tissue and organ involvement, which can lead to significant diagnostic delays.^86^ ^87^ Unlike LHON which tends to be monosymptomatic with no impact on life expectancy, a subset of patients harbouring more deleterious mtDNA mutations or nuclear genetic defects that result in severe mtDNA depletion can develop an aggressive disease course, frequently starting in early childhood, and characterised by irreversible encephalopathy, intractable epilepsy, liver failure and multisystem organ failure. The outcome of these mitochondrial syndromes is invariably fatal and in the absence of effective treatments, significant effort has been invested in developing tractable means of selectively eliminating these pathogenic mutations through germline genome editing, or in preventing the maternal transmission of pathogenic mtDNA mutations from mother to child.^92^

### Germline genome editing

There are about 2300 women of childbearing age in the UK harbouring pathogenic mtDNA mutations and by using the national fertility rate, nearly 150 pregnancies per year could result in the birth of a child at high risk of developing severe mitochondrial disease.^93^ Genetic counselling for prospective mothers harbouring heteroplasmic mtDNA mutations remains challenging as there can be rapid shifts in mitochondrial allele frequencies due to the ‘mitochondrial bottleneck’ operating in the early stages of oocyte development.^94–96^ As the majority of mtDNA mutations cause disease when the level of heteroplasmy exceeds 70–80%, preimplantation genetic diagnosis (PGD) could be used to select the woman's embryo carrying the lowest mutant load, and therefore most likely to result in a healthy child.^97^ However, there is only limited clinical experience in the use of PGD for mitochondrial diseases and there is always the risk that the mutation load detected in biopsied blastomeres or trophectoderm does not accurately represent the entire embryo, or more importantly, the level in the tissue most at risk from a particular mtDNA mutation.^98^ To circumvent these difficulties, several research groups worldwide are working on mitochondrial-targeted nucleases that have been engineered to selectively eliminate mutated mtDNA molecules.^99–101^

**Figure 2:**
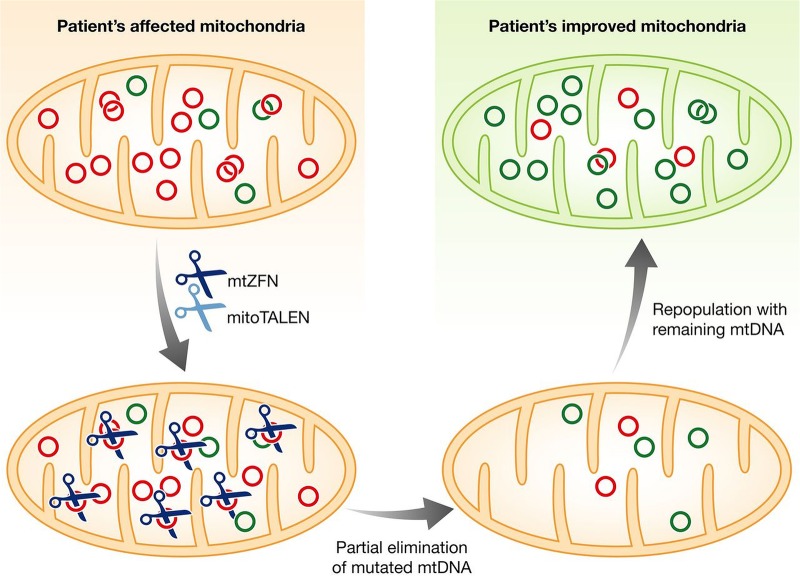
Elimination of mutated mtDNA molecules with specifically designed mitochondrial nucleases. Zinc finger nucleases (ZFNs) and transcription activator-like effector nucleases (TALENs) first need to be targeted to the mitochondrial matrix compartment where they can physically interact with mtDNA molecules. There are a number of possible strategies, including transfecting the cell with plasmid vectors, the use of viral vectors to deliver the gene construct or direct injection of mRNA encoding for the nuclease. The use of transiently expressed RNA in oocytes or one-cell embryos circumvents the disadvantages of exogenous DNA administration in the germline, which remains a cause of concern with other mitochondrial replacement techniques such as pronuclear transfer and metaphase II spindle transfer. ZFNs and TALENs can be designed to recognise specific DNA sequences and they have the ability to create double-strand breaks that eliminate mutated mtDNA molecules. There is a transient reduction in mtDNA copy number, but the remaining mtDNA molecules are able to proliferate, repopulating the mitochondria with a higher proportion of the wild-type species. The end result is a beneficial reduction in the cell's overall mutant load and an improved bioenergetic profile. Adapted from Moraes.^100^

The principle is straightforward and it makes use of the differences in restriction sites created by the mtDNA mutation (figure 2). Zinc finger nucleases (ZFNs) and transcription activator-like effector nucleases (TALENs) are able to recognise these altered DNA sequences and they create double-strand breaks that effectively eliminate the mutated mtDNA molecules.^102–106^ The ability to shift the level of heteroplasmy could be used to reduce the overall mutant load in the oocyte of a woman carrying a known pathogenic mtDNA mutation to subthreshold level, thereby eliminating the risk of her child developing overt mitochondrial disease. The use of ZFNs or TALENs as a reproductive tool for manipulating levels of heteroplasmy is still in early stages of development and a number of technical difficulties need to be resolved.^99–101^ There are still issues about the best way of delivering these nucleases to the mitochondrial matrix and safety concerns need to be addressed further due to the possibility of deleterious off-target effects in the nuclear genome. Germline genome editing, namely with the CRISPR/Cas9 system, could also be used to correct for pathogenic mutations within the nuclear genome.^107–109^ This technology is attracting significant interest (and debate) within the scientific community and also within the wider public, as genetic manipulation of the germline and experimentation on early human embryos raises a number of important ethical and legal considerations.^110–112^

### Mitochondrial replacement

**Figure 3:**
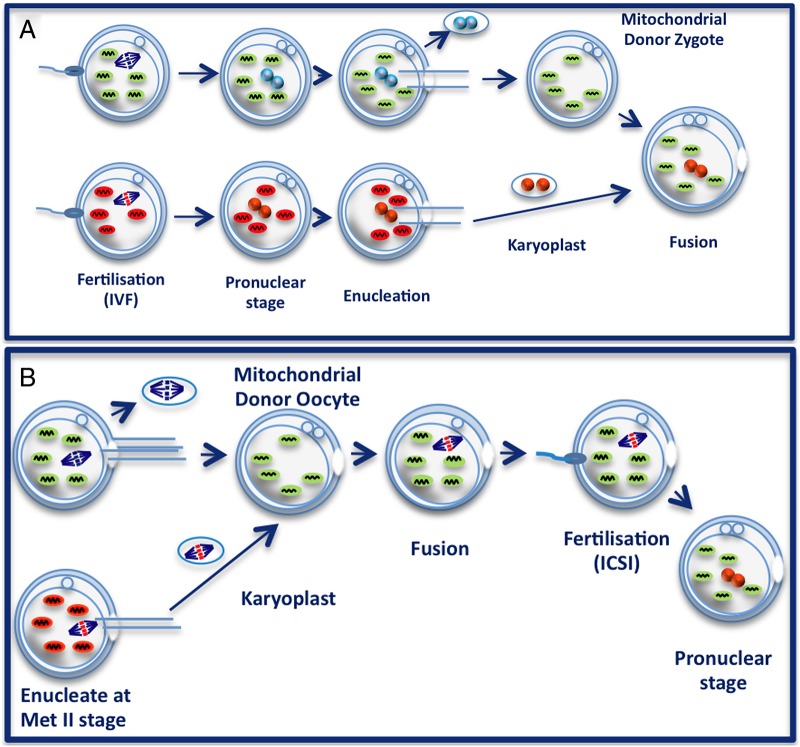
IVF methods to prevent the maternal transmission of mtDNA mutations. (A) Pronuclear transfer. An embryo is created via conventional in vitro fertilisation using the father's sperm and the mother's oocyte, the latter carrying a homoplasmic mtDNA mutation (red mitochondria). After fertilisation has taken place, the parental pronuclei (red circles) are removed from the single-cell embryo and they are then transferred into a mitochondrial donor zygote harbouring only wild-type mtDNA (green mitochondria). (B) Metaphase II spindle transfer. The maternal spindle is a structural unit that packages the mother's nuclear DNA in the unfertilised oocyte. In this alternative approach, the intended mother's metaphase II spindle is transferred into a mitochondrial donor oocyte, and this is then followed by intracytoplasmic sperm injection (ICSI) fertilisation (http://blog.wellcome.ac.uk/2012/01/20/a-good-concept-science-mitochondrial-dna/, accessed on 18 December 2015).

The elimination of mutated mtDNA molecules to shift the level of heteroplasmy to subthreshold level is an attractive strategy to prevent a biochemical deficit and rescue the cellular phenotype. However, in some mitochondrial diseases, such as LHON, the majority of carriers harbour homoplasmic mtDNA mutations and a different experimental strategy is needed to prevent the transmission of a pathogenic mtDNA mutation from mother to child.^56^ ^57^ Two related in vitro fertilisation (IVF) techniques have been developed that involves transferring the parental nuclear genetic material into a donor cytoplast containing a normal wild-type mtDNA population (figure 3A, B).^113–115^ There is minimal carryover of mutant mtDNA (<2%) with pronuclear transfer and metaphase II spindle transfer, and both methods have been shown to be compatible with normal embryonic development and the birth of healthy offspring in a nonhuman primate model. Although encouraging, further work is needed to explore the safety implications of these IVF techniques for embryo development, including the concerns that have been raised about epigenetic abnormalities and the possibility of nuclear–mitochondrial genetic mismatch leading to unforeseen negative consequences.^116^ ^117^

If mitochondrial replacement is adopted for the prevention of mitochondrial disease, the child's entire genetic make-up will be derived from the biological parents except for the 37 mitochondrial genes inherited from the female donor oocyte.^118^ However, mitochondrial replacement carries important long-term implications as it involves germline manipulation transmissible to future generations.^119^ There is, understandably, fierce debate on this topic and a comprehensive public consultation exercise involving all the major stakeholders was initiated in the UK to discuss not only the scientific merits, but also the possible ramifications to society as a whole. The Nuffield Council on Bioethics has concluded that mitochondrial replacement might be appropriate within a strictly regulated research environment, and with the prospective parents being fully informed about the potential risks, both real and theoretical.^120^ In February 2015, both Houses of Parliament in the UK have voted strongly in favour of mitochondrial donation to prevent the maternal transmission of mitochondrial disease (http://www.parliament.uk/business/news/2015/february/lords-mitochondrial-donation-si/, accessed on 18 December 2015). The clinical application is expected to begin within the next 2 years and if approved, this procedure will be closely monitored by the Human Fertilisation and Embryology Authority (HFEA, UK).

## Conclusion

The launch of the Human Genome Project in 1990 was a seminal moment in the history of science and it started a rapid expansion in technology that continues unabated today. More recently, the availability of whole-exome and whole-genome sequencing in routine clinical practice has accelerated gene discovery and the genetic basis for the vast majority of monogenic diseases will likely be uncovered within the next 5–10 years. There are still limited treatments for most inherited neurodegenerative disorders and the next crucial step now is to translate this genomic revolution into tangible benefits for patients and their families. Gene therapy has had many setbacks over the years, but this field of research has matured, and there is now a much better understanding of gene delivery systems and the pathological pathways that could be manipulated to minimise disease burden. There is a still long way to go as the development of gene therapy for the central nervous system is far more challenging than for inherited retinal diseases, which benefit from the eye's relative ease of access and immune privilege. Germline genome editing is an exciting technological development that has the potential to prevent the transmission of both nuclear and mtDNA mutations from mother to child, but safety issues need to be rigorously addressed before it can be considered for clinical application. There is also the need for a wider debate within society about the ethical, moral and legal implications of manipulating the germline in human embryos, and the framework that will need to be put in place to avoid any misuse. Genetic manipulation for inherited neurodegenerative diseases is certainly not a myth, but a considerable amount of work is still needed before it becomes a reality.
